# USING DATA SCIENCE TO GENERATE PSYCHOSOCIAL PROFILES OF FINANCIAL EXPLOITATION IN SENIORS

**DOI:** 10.1093/geroni/igz038.1780

**Published:** 2019-11-08

**Authors:** Cassandra J Enzler, Robert Suchting, Charles Green, Jason Burnett

**Affiliations:** University of Texas Health McGovern Medical School, Houston, Texas, United States

## Abstract

Financial exploitation (FE) in older adults is a significant public health problem linked to outcomes including depression, financial ruin and early mortality. This study applied exploratory data science techniques to a multi-year statewide protective services dataset of over 8,000 elder abuse cases. The goal was to derive data-driven psychosocial profiles of abuse with an emphasis on determining which factors, commonly shared across abuse cases, were most important for determining when elder FE was occurring and whether it was occurring alone or in conjunction with other types of abuse. We found that pronounced psychological distress (i.e. verbalizing suicide, homicide, self-harm) was most important for indicating when abuse had occurred and predicted non-FE related abuse. Drug paraphernalia in the home and perpetrator drug/alcohol use were important predictors of FE-related abuse. When differentiating pure FE from hybrid FE, factors indicative of long-term FE occurrence and substantial financial loss were most important (i.e. facing foreclosure, lack of food, medications, and utilities). The findings parallel some existing work characterizing pure and hybrid FE, but also highlight new profile factors that may help determine when FE is occurring and when it is less likely. Applying data science approaches to other large protective service datasets and national datasets such as the National Adult Maltreatment Registry could help improve characterization of abuse types such as pure and hybrid FE resulting in better detection, response and prevention.

